# New journal structure of child and adolescent psychiatry and mental health

**DOI:** 10.1186/s13034-016-0123-6

**Published:** 2016-09-23

**Authors:** Joerg M. Fegert, Benedetto Vitiello

**Affiliations:** 1 University Hospital Ulm, Ulm, Germany; 2 National Institute of Mental Health, Bethesda, USA

In June 2007, the first four manuscripts were published in Child and Adolescent Psychiatry and Mental Health (CAPMH). In its early years, CAPMH was the first open access online journal in the field of child mental health. This innovative idea was funded by a grant from the German Research Foundation (Deutsche Forschungsgemeinschaft, DFG) for several years. To date, 9 years later, CAPMH has proven to be a success story by having reached an official Thomson Reuters impact factor of 2.134. This seems to be the right moment to describe achievements of CAPMH over the past years, and to also take a look into the future of a new structure of CAPMH.

The European Union Competitiveness Council (comprised of science, innovation, trade and industry ministers of the 28 member states) announced an update on HORIZON 2020 in May 2016, agreeing to “support a transition to immediate open access as the default by 2020, using the various models possible and in a cost-effective way, without embargoes or with as short as possible embargoes, and without financial and legal barriers, taking into account the diversity in research systems and disciplines.”, meaning that the results of publically funded research should be free for anyone to access and re-use the data, and not be placed under embargo. The Council’s recommendations are not legally binding, however a strong recommendation towards open access is clearly evident, with an aim to have all scientific articles freely accessible by 2020. According to Web of Science, there has been a steady growth of open access publications over the last view years in the fields of psychiatry and psychology. Along with this general growth of online publications, CAPMH has seen a steady growth of submissions and publications over the years. While 16 manuscripts were published in Volume 1 in 2007, 55 were published in 2015. The average time from acceptance to publication in 2016 was on average 15 days. This fast turnaround can be achieved by the online structure of the journal, in which manuscripts are not constrained to be published within issues. Most highly cited publications since 2013 as stated by Web of Science included a study on suicidal behaviors in adolescents from France [1] and a study among psychologists about diagnosing personality disorders in adolescents from the Netherland [2].

The journal has benefited from cooperating with several organizations throughout the past years. Since 2013, CAPMH has been the official journal of the *International Association of Child and Adolescent Psychiatry and Allied Professions* (IACAPAP). In cooperation with IACAPAP, the *European Association for Forensic Child and Adolescent Psychiatry, Psychology and other involved Professions* (EFCAP) and the *International Society for the Study of Self Injury* (ISSS), several special series have been published, including some of the most highly accessed manuscripts on CAPMH.

CAPMH has always aimed to publish high quality clinical and research papers from around the world. Support of unusual research initiatives from low- to middle income countries, as well as international, interdisciplinary networking initiatives are especially important to the journal culture of CAPMH. For example, a study on nail biting and psychiatric disorders in children from Iran [3] has been accessed almost 20,000 times. As an online open access journal, CAPMH reaches allied professions i.e. from the forensic field, such as criminologists or other legal professions. The same holds true for other occupations in the field of child mental health, such as social workers or teachers.

We would like to thank the members of the Editorial Board, who have supported the development of CAPMH throughout the years. After receiving the Impact Factor, the editorial team is now faced with new challenges, which call for the need of restructuring the journal. Special thanks go to Prof. Christian Kieling, who has served as an Associate Editor from South-America for many years and has also facilitated the liaison with IACAPAP. We would also like to thank all members of the Editorial Board who are stepping down and are looking forward with our new and remaining members.

A warm welcome goes to our new Associate Editors: Alka Ahuja, Rebecca Groschwitz, Takahiko Inagaki, Ferdinand Keller, Kavitha Pasunuru, and Paul Plener, as well as our Liaison Editor for EFCAP Cyril Boonmann. A new Liaison Editor for IACAPAP will be nominated at IACAPAP, Calgary Meeting in September 2016. Special thanks for their continuous support the journal goes the senior editors of CAPMH Jacinta Tan Jacinta Tan, Lutz Goldbeck, and Lim Choon Guan.

We are looking forward to many more productive years of CAPMH and would like to invite all researchers and clinicians in the field to submit their valued work to the journal.

Prof. Joerg M. Fegert (Editor-in-Chief) and Dr. Benedetto Vitiello (Deputy Editor).
